# Identification of a New Ribonucleoside Inhibitor of Ebola Virus Replication

**DOI:** 10.3390/v7122934

**Published:** 2015-12-02

**Authors:** Olivier Reynard, Xuan-Nhi Nguyen, Nathalie Alazard-Dany, Véronique Barateau, Andrea Cimarelli, Viktor E. Volchkov

**Affiliations:** 1 Molecular Basis of Viral Pathogenicity, CIRI, INSERM, U1111-CNRS UMR5308, Université de Lyon, Université Claude Bernard Lyon 1, Ecole Normale Supérieure de Lyon, Lyon 69007, France; olivier.reynard@inserm.fr (O.R.); nathalie.alazard-dany@inserm.fr (N.A.-D.); 2 Host Pathogen interaction during lentiviral infection, CIRI, INSERM, U1111-CNRS UMR5308, Université de Lyon, Université Claude Bernard Lyon 1, Ecole Normale Supérieure de Lyon, Lyon 69007, France; xuan-nhi.nguyen@ens-lyon.fr (X.-N.N.); vbarateau@ens-lyon.fr (V.B.) acimarel@ens-lyon.fr (A.C.)

**Keywords:** Ebola, Marburg, Filovirus, polymerase, antiviral, inhibitors

## Abstract

The current outbreak of Ebola virus (EBOV) in West Africa has claimed the lives of more than 15,000 people and highlights an urgent need for therapeutics capable of preventing virus replication. In this study we screened known nucleoside analogues for their ability to interfere with EBOV replication. Among them, the cytidine analogue β-d-N4-hydroxycytidine (NHC) demonstrated potent inhibitory activities against EBOV replication and spread at non-cytotoxic concentrations. Thus, NHC constitutes an interesting candidate for the development of a suitable drug treatment against EBOV.

Highlights: (1) A novel inhibitor of Ebola virus replication is identified; (2) 2 µM treatment is sufficient to inhibit EBOV spread *in vitro.*

West Africa is currently undergoing the largest outbreak of Ebola virus (EBOV) ever described, a crisis that has claimed an heavy toll in terms of human life [1] and has strongly affected the African economy. The acute character of the infection, the extremely high lethality of EBOV infection and an absence of approved therapeutics make it imperative to gather a large spectrum of antiviral approaches that can be used to counter virus replication. EBOV belong to the *Filoviridae* family, that comprise a group of enveloped negative-strand RNA viruses responsible for severe haemorrhagic fever in humans [2]. The EBOV genome is about 19 kb long and codes for seven structural proteins and several non-structural proteins [3,4,5,6]. The polymerase L, responsible for synthesis of viral RNAs [7] is the most obvious target for antivirals as exemplified by the currently field-tested Favipiravir [8].

In this study several nucleoside analogues were tested for their ability to interfere with thereplication of EBOV (Table 1). Among them: 2’,3’-didehydro-3’-deoxythymidine (Stavudine) and 3’-azido-3’-deoxythymidine (Zidovudine), both of which are nucleoside analogues of thymidine; phosphonic acid (((1*R*)-2-(6-amino-9H-purin-9-yl)-1-methylethoxy) methyl)-monohydrate (Tenofovir)—an adenosine analogue; four cytidine nucleoside analogues: 2’,3’-dideoxcytidine (ddC), 2’,3’-dideoxy, 3’-thiacytidine (Lamivudine), 5-fluoro-1-(2*R*,5*S*)-(2-hydroxymethyl)-1,3-oxathiolan-5-yl) cytosine (Emtricitabine) and β-d-*N*^4^-hydroxycytidine (NHC); (1*S*,*cis*)-4-(2 amino-6-(cyclopropylamino)-9H-purin-9-yl)-2-cyclopentene-1-methanol (Abacavir) and 2’,3’-dideoxyinosine (ddIi), that are guanosine analogues. In addition, hydroxyurea (HU), an inhibitor of the ribonucleotide reductase was also tested as potential inhibitor of EBOV replication. Some of these compounds are currently used in the clinical practice in the management of HIV-1 infection [9,10,11,12], while NHC has been described to possess antiviral activity *ex vivo* against different RNA viruses [13].

**Table 1:** List of compounds used here.

Compound	Chemical Name
**Thymidine Analogues**	
Stavudine	2’,3’-didehydro-3’-deoxythymidine
Zidovudine	3’-azido-3’-deoxythymidine
**Adenosine Analogue**	
Tenofovir	phosphonic acid (((1*R*)-2-(6-amino-9H-purin-9-yl)-1-methylethoxy) methyl)-monohydrate
**Cytidine Analogues**	
ddC	2’,3’-dideoxcytidine
Lamivudine	2’,3’-dideoxy, 3’-thiacytidine
Emtricitabine	5-fluoro-1-(2*R*,5*S*)-(2-(hydroxymethyl)-1,3-oxathiolan-5-yl) cytosine
NHC	β-d-*N*^4^-hydroxycytidine
**Guanosine Analogues**	
ddI	2’,3’-dideoxyinosine
Abacavir	(1*S*,*cis*)-4-(2 amino-6-(cyclopropylamino)-9H-purin-9-yl)-2-cyclopentene-1-methanol
**RNR Inhibitor**	
HU	hydroxyurea

To evaluate the potential antiviral effect of the above-mentioned drugs, Vero E6 cells were infected at a multiplicity of infection (MOI) of 0.1 and maintained in Dulbecco’s Modified Eagle Medium (DMEM) supplemented with 3% foetal calf serum (FCS) in the presence of the compounds provided at a concentration of 10 μg/mL (~30–50 µM), except HU that was instead used at 1 mM. For the initial screen the extent of viral replication was assessed at a single time point (48 h post infection). A recombinant EBOV carrying an additional transcription cassette between *VP35* and *VP40* genes and expressing enhanced green fluorescent protein (eGFP) (EboV/GFP) was used, as described elsewhere [14]. A similar approach have been used recently by Albarino *et al.* [15], and have proven to be useful and reliable in drug screening. Notably EBOV/GFP virus allows simultaneous measurement of two important parameters during viral infection by flow cytometry. First, since GFP reporter is encoded within the genome of the recombinant virus, a precise measurement of viral spread is possible through the determination of the percentage of GFP-positive cells. Second, since the GFP expression is driven by the viral polymerase, EBOV transcription efficacy can be assessed by measuring the Mean Fluorescent Intensity (MFI) of the GFP signal. Under these conditions, only NHC and HU displayed a notable activity against EBOV/GFP replication, among the compounds tested here (Figure 1). NHC induced a significant drop in the number of infected cells, as well as in the intensity of GFP expression (5 fold and 2.5 fold inhibition, respectively). On the contrary, HU induced a 1.5 fold decrease in GFP MFI, but did not reduce virus spread. For these reasons our further efforts were concentrated on NHC.

The ability of NHC to interfere with viral replication as well as its potential cytotoxicity were evaluated over a wide concentration range in both VeroE6 cells and primary macrophages. Primary macrophages that represent a key cellular target for EBOV replication *in vivo* [16] were obtained upon macrophage colony stimulating factor M-CSF treatment of blood monocytes according to a well-established procedure [17]. Vero E6 cells (70%–80% confluent) and macrophages (three different blood donors) were treated with NHC at different concentrations in the range of doses comprised between 1.5 and 48 µM and were infected with EBOV/GFP prior to flow cytometry analysis 48 h afterwards (Figure 2A,B, respectively). The analysis of the compound potency on Vero E6 cells revealed that NHC half maximal effective concentration (EC_50_) was 3 and 3.8 µM for inhibition of transcription and virus spread, respectively (calculated using Graphpad Prism software, La Jolla, CA, USA). NHC was also highly effective in interfering with EBOV replication in monocyte-derived macrophages, while being slightly less efficient than in Vero E6 cells in two out of three donors (Figure 2B). MTT cytotoxicity assay performed on both Vero E6 cells and macrophages revealed no significant effects on cell viability after a 48 h incubation with NHC and when the inhibitor was used at concentration below 12 µM (Figure 2C). At higher concentrations (24 and 48 µM), NHC displayed a moderate cytotoxicity, although in the absence of cell detachment and cell rounding. These data are in line with published reports on the absence of significant toxicity caused by the inhibitor at the concentration below 75–100 µM in Madin-Darby canine kidney cells (MDCK), human hepato cellular carcinoma cells (HUH), HepG2 cells or *ex vivo in* human peripheral blood monocytes [13], as well as in a mouse model where NHC was provided up to 126 mM per kilogram of body weight during five consecutive days without any measurable side effects [13].

**Figure 1:**
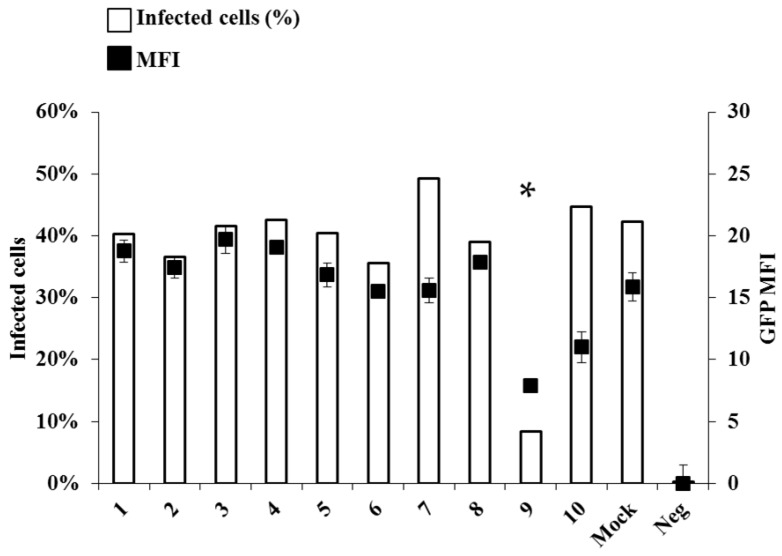
Screening of antiviral compounds against Ebola virus (EBOV). Vero E6 cells were infected with EBOV expressing GFP (EBOV/GFP) at an multiplicity of infection (MOI) of 0.1 in the presence of different chemical compounds (see Table 1) at the indicated concentration. Forty-eight hours afterwards, cells were detached from the plate with trypsin and fixed for 20 min with 3% PBS buffered paraformaldehyde prior to flow cytometry analysis on a Beckman Coulter Gallios apparatus (Beckman, Brea, CA, USA). Neg value indicate the background level in the non infected condition. The graph presents both the % of GFP-positive cells, reflecting the extent of viral spread and the median fluorescence intensity (MFI), simulating the level of viral transcription. All data represents averages and SEM of three independent experiments. (Please replace by: All data represents averages and SD of three independent experiments. * A student test was performed between value of compound 9 and the Mock treated condition, *p*-value below 0.05. The data are representative of the values of two different experiments performed in triplicate).

Next, in order to exclude a possible non-specific effect of NHC on GFP expression, Vero E6 cells were transfected with a plasmid DNA expressing GFP and after 6 h the cells were treated with NHC (1.9 or 7.2 µM) and then incubated for additional 40 h prior to flow cytometry. No differences in GFP expression were observed between treated *versus* untreated cells, indicating that NHC did not exert non-specific effects on the GFP reporter *per se* (Figure 3A).

In attempt to better understand at which step NHC interferes with the viral replication, Vero E6 cells were infected at an MOI of 0.1 for one hour, washed twice with DMEM, incubated in DMEM 3% FCS with or without NHC (1.9 and 7.2 µM) and then the accumulation of anti-genomic and genomic RNAs was quantified using qRT-PCR. Under these conditions, both genome and anti-genome synthesis was similarly impaired by NHC treatment indicating that NHC have a global effect on viral RNA replication (Figure 3B).

**Figure 2:**
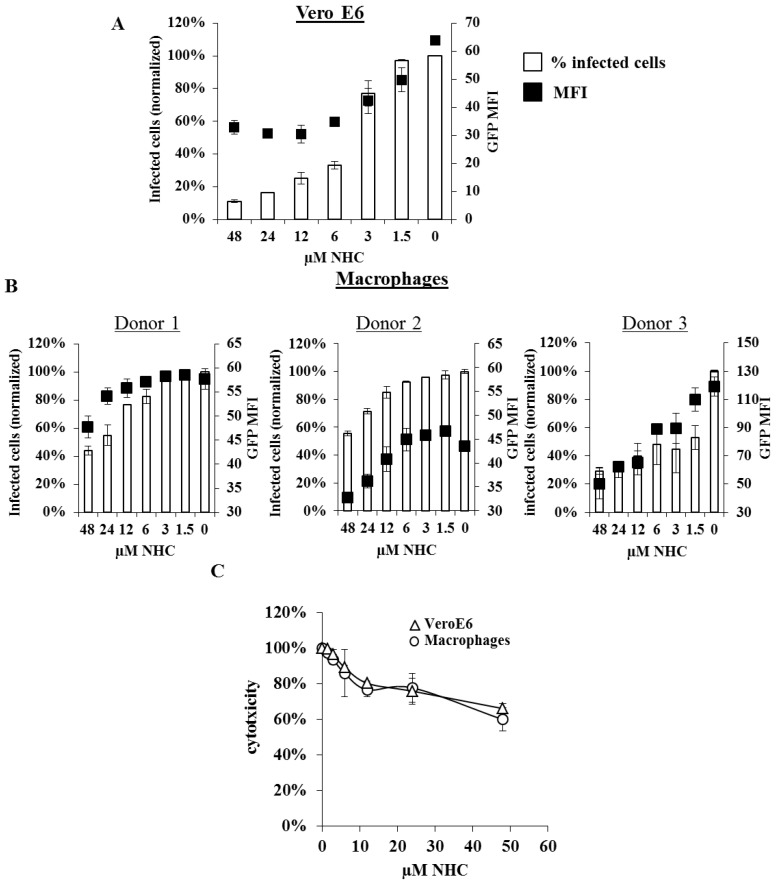
Dose-response assessment and β-D-N4-hydroxycytidine (NHC) cytotoxicity Vero E6 cells (**A**) and monocyte-derived macrophages (**B**) were infected with EBOV/GFP at an MOI of 0.1, and maintained in the presence of NHC at the indicated concentrations. The cells were analyzed forty-eight hours post infection as described in Figure 1. Macrophages were differentiated upon incubation of monocytes (three different donors) with 100 ng/mL of M-CSF (EuroBio, Courtaboeuf, France). The graph displays both the percentage of infected cells and the GFP MFI as measured by flow cytometry (Beckman Coulter, Gallios, Brea, CA, USA); (**C**) MTT (3-(4,5-dimethylthiazol-2-yl)-2,5-diphenyltetrazolium bromide) cytotoxicity assay. Vero E6 cells and monocyte-derived macrophages were maintained in the NHC presence for 48 h prior to addition of MTT (5 µg/mL, Sigma Aldrich, Saint-Quentin Fallavier, France) followed by 3 h incubation and optical density analysis The graph displays the OD value normalized to the 100% cytotoxicity control.

**Figure 3:**
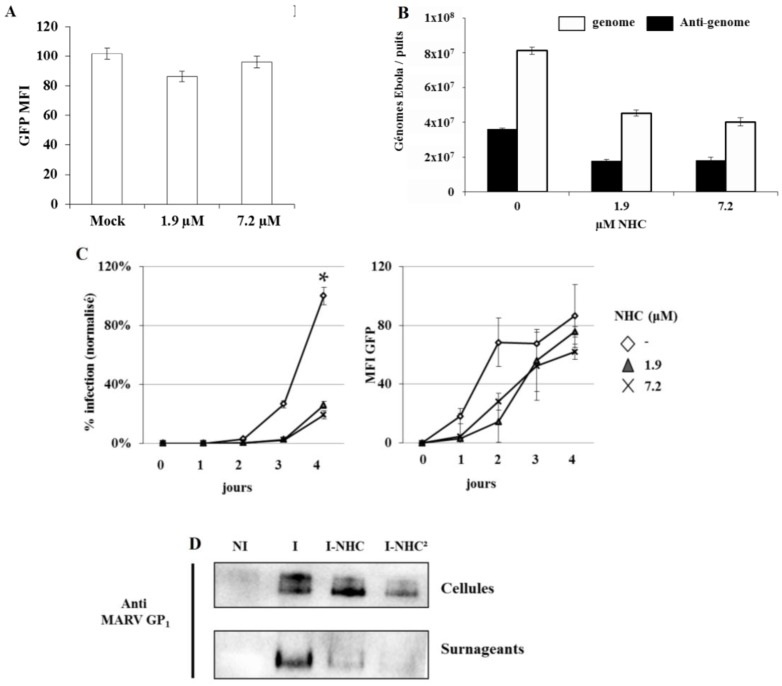
Mode of NHC antiviral activity: (**A**) NHC effect on expression of GFP. Vero E6 cells were transfected with 3 µg of plasmid DNA expressing GFP (peGFP N1, Clontech) using FugeneHD (Promega), GFP MFI was measured on a Becton Dickinson, LSRII. The graph presents averages and SEM of three independent experiments; (**B**) qRT-PCR analysis of genomic and anti-genomic RNA. Vero E6 cells were infected with EBOV/GFP at an MOI of 0.1 and analyzed 24 h post infection by qRT-PCR. The reverse transcription Iscript select cDNA synthesis (Biorad) was followed by qPCR (Faststart universal Sybr green and LC96 apparatus, both Roche diagnostics) using forward and reverse primer in the non-transcribed trailer region (TR-F: 5’ATTACTGCCGCAATGAATTTAACGC (18429), TR-R: 5’AACAATATGAGCCCAGACCTTTCG (18517)). To distinguish between genomic and antigenomic RNAs, the reverse transcription step was carried out using either a forward or a reverse primer, respectively. The data are representative of the values of two separate experiments performed in duplicate. Data were normalized by using GAPDH Fw and Rev primer (5′CACCCACTCCTCCACGTTTGAC and 5′GTCCACCACCCTGTTGCTGTAG respectively); (**C**) Inhibition of EBOV replication and spread by NHC. Vero E6 cells were infected with EBOV/GFP at an MOI of 0.01 and were analyzed daily by flow cytometry. **Left panel**: present of infected cells; **right panel**: relative GFP fluorescent intensity. The data are representative of the values of two different experiments performed in duplicate. * A student test was performed between value of treated and the Mock treated condition at day 4, *p*-value below 0.05 for both 1.9 and 7.2 µM; (**D**) Effect of NHC on Marburg virus replication. Vero E6 cells were infected with Marburg virus (Musoke isolate) at MOI 0.1 and treated with NHC (7.2 µM) for a period of four days prior to WB analysis. NI, non-Infected cells; I, infected cells; I-NHC, infected cells treated with NHC at day 0; I-NHC^2^, infected cells treated with NHC twice. Second treatment was performed at day 2 after cell washing and media replenishment. Western blot analysis of cells and culture supernatants was made using a mouse anti-Marburg GP1 antibody (clone 6589). The data are representative of the values of two different experiments.

Next, the effects of NHC were monitored daily over 4 days both for the virus spread as well as for the efficiency of GFP expression (Figure 3C). Vero E6 cells were infected with EBOV-GFP at an MOI of 0.01 and were analysed daily by flow cytometry. Approximately 20% of cells became GFP-positive by day 3 post infections in the absence of treatment. In contrast, only approximately 1.6% of cells were GFP-positive in the presence of NHC. The MFI analysis indicated that NHC robustly reduced EBOV transcription within the first two days after infection (Figure 3C). At later time point (4 days post infection), approximately 18% of NHC-treated cells displayed expression of GFP indicating a delay rather than a complete blockage of EBOV replication caused by NHC treatment. In agreement with this hypothesis, the MFI of cells infected in the presence of NHC increased by day 2 post infection, likely preceding the increase in viral spread observed by day 4. One possible explanation could be the emergence of NHC-resistant virus variants. Each of four consecutive passages of EBOV in the presence of the inhibitor showed a similar pattern of the virus growth as seen during initial virus passage. Moreover, a reduction in virus titers observed after each consecutive virus passage diminished the possibility of further passaging. Worthy to notice that attempts to generate NHC-resistant variants of HCV also failed in the study of Stuyver *et al.* [13].

Next we investigate whether NHC was effective against Marburg virus (MARV), another Filoviridae family member. For this purpose, VeroE6 cells were infected with Marburg virus (Musoke isolate) at an MOI of 0.1 and treated or not treated with NHC at 7 µM. In the absence of the MARV expressing GFP virus, replication and spread in presence of NHC, were analysed by Western blot using surface glycoprotein GP specific antibodies (Figure 3D). A treatment with NHC diminished the amounts of MARV GP in both cell lysates and supernatants, indicating that this other member of the Filoviridae family are also sensitive to NHC. In a separate experiment, MARV-infected cells were washed 2 days post infection and then replenished with a medium containing fresh NHC for further 2 days (7 µM). Under these conditions, an even more apparent effect on viral replication was observed, indicating that repetitive NHC treatment increases drug efficacy.

An alternative explanation to the fact that EBOV spread is effectively blocked early post infection, but less so at later time points could be that the compound is readily metabolized within the cells. In effect such hypothesis was postulated also in other studies where the authors demonstrated that NHC could be rapidly phosphorylated and metabolized to the cytidine and uridine mono-, di- and three-phosphates (MPs, DPs, and TPs) [13,18].

The mechanism of NHC antiviral action is not fully understood while this compound has been described to possess antiviral activity *ex vivo* against different RNA viruses [13]. Several possible modes of NHC action have been proposed notably: (i) a direct interaction with viral proteins; (ii) an interaction with host cell proteins; or (iii) an indirect effect through the incorporation and disruption of important secondary/tertiary structures of viral RNAs. Earlier it had been demonstrated that while not directly inhibiting the polymerase of HCV, NHC is capable to interfere with cytoplasmic RNA metabolism in HCV-infected cells [13]. Potentially NHC may act as an antimetabolite, similarly to ribavirin. This mode of action may result in an inhibition of cellular Inosine-5’-monophosphate dehydrogenase resulting in a dramatic reduction of GTP and dGTP intracellular concentration [19]. In this respect, a compound closely related to NHC, the 5-monophosphates *N*(4)-hydroxy-2-deoxycytidines was found to be a potent inhibitor of thymidylate synthase [20]. The third possible mode of NHC action could be explained by ability of this compound to act as a weak alternative substrate for CTP. In this case, when incorporated into the viral RNAs NHC could change the thermodynamics of RNA secondary structures leading to a potential modulation of virus replication steps that are dependent on those secondary structures, such as for example encapsidation, translation or replication. While the role of secondary structures in filoviruses replication is less obvious due to a tight encapsidation of genomic RNA by the nucleoprotein NP, it is intriguing that all viral mRNAs contain particular secondary structures that include conserved transcription initiation signals [21,22]. Furthermore, earlier it had been shown that both genomic and anti-genomic promoters of both filoviruses contain sequences capable to form stable secondary structures [23]. NHC may also behave as a mutagenic substrate for the L polymerase. Indeed, it had been shown that triphosphorylated NHC could be incorporated in viral genome potentially introducing mutations during RNA replication as in such case NHC behave both as cytosine or uracil [13].

In conclusion, in our study we demonstrate that NHC is potentially an effective antiviral compound inhibiting Ebola and Marburg virus replication *in vitro,* thus warranting further studies, in particular the analysis of NHC derivatives of increased stability and antiviral efficacy. Intriguingly, low NHC toxicity in mice suggests its potential for being used *in vivo*. The development and use of NHC along with other antiviral strategies may provide an important tool to control early stages of EBOV infection and to restrain virus spread.
