# Correction: Reynard, O.; *et al*. Identification of a New Ribonucleoside Inhibitor of Ebola Virus Replication. *Viruses* 2015, 7, 6233‒6240

**DOI:** 10.3390/v8050137

**Published:** 2016-05-18

**Authors:** Viruses Editorial Office

**Affiliations:** MDPI AG, Klybeckstrasse 64, CH-4057 Basel, Switzerland

The *Viruses* Editorial Office wishes to notify its readers of corrections in [1]. 

The authors refer to [2], writing that the experiments were done using 126 mM/kg/day for mouse treatment without side effects. The correct dose used in [2] was 126 µmol/kg/day.

On page 6235, the last sentence of the first paragraph should read: “These data are in line with published reports on the absence of significant toxicity caused by the inhibitor at the concentration below 75–100 M in Madin-Darby canine kidney cells (MDCK), human hepato cellular carcinoma cells (HUH), HepG2 cells or *ex vivo* in human peripheral blood monocytes [13], as well as in a mouse model where NHC was provided up to 126 µmol per kilogram of body weight during five consecutive days without any measurable side effects [13]”.

The original Figure 3 was in French and has been replaced by the following:

**Figure:**
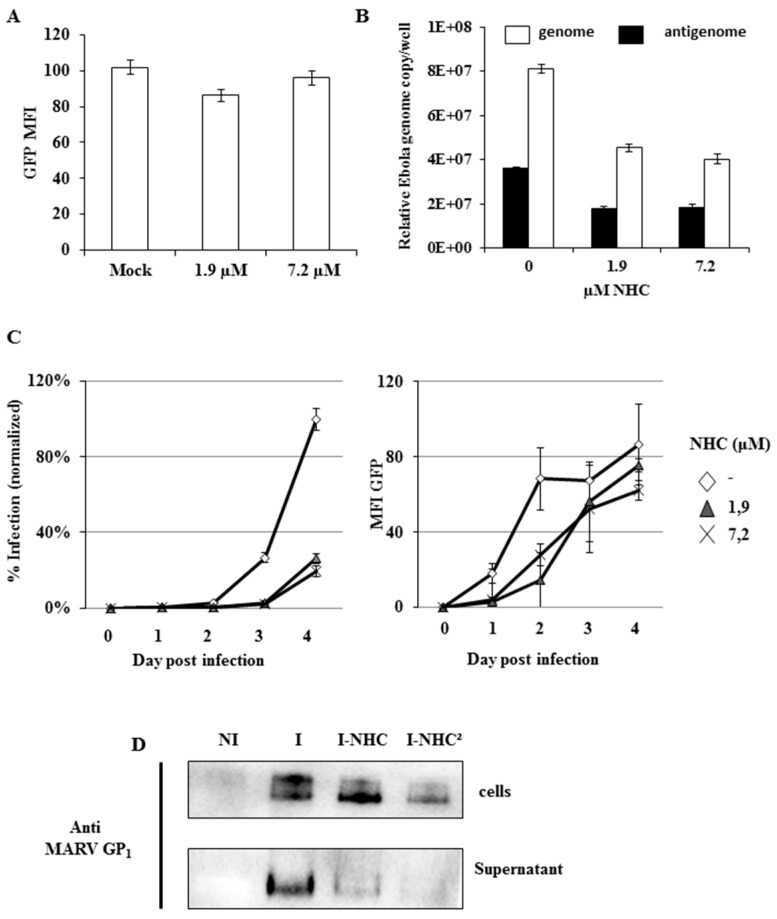


The manuscript will be updated and the original will remain available on the article webpage. The authors would like to apologize for any inconvenience caused.
